# Clues to the Design of Aggregation-Resistant Insulin from Proline Scanning of Highly Amyloidogenic Peptides Derived from the N-Terminal Segment of the A-Chain

**DOI:** 10.1021/acs.molpharmaceut.4c00077

**Published:** 2024-03-25

**Authors:** Wojciech Puławski, Robert Dec, Wojciech Dzwolak

**Affiliations:** † Bioinformatics Laboratory, Mossakowski Medical Research Institute, Polish Academy of Sciences, Pawinski Street 5, 02-106 Warsaw, Poland; ‡ Faculty of Chemistry, Biological and Chemical Research Centre, University of Warsaw, Pasteur Street 1, 02-093 Warsaw, Poland

**Keywords:** insulin aggregation, amyloid, insulin receptor, proline scan, A-chain

## Abstract

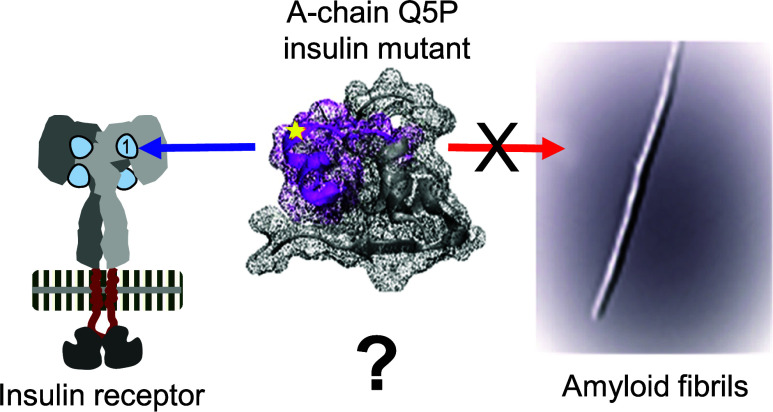

Insulin aggregation poses a significant problem in pharmacology and medicine as it occurs during prolonged storage of the hormone and *in vivo* at insulin injection sites. We have recently shown that dominant forces driving the self-assembly of insulin fibrils are likely to arise from intermolecular interactions involving the N-terminal segment of the A-chain (ACC_1–13_). Here, we study how proline substitutions within the pilot GIVEQ sequence of this fragment affect its propensity to aggregate in both neutral and acidic environments. In a reasonable agreement with *in silico* prediction based on the Cordax algorithm, proline substitutions at positions 3, 4, and 5 turn out to be very effective in preventing aggregation according to thioflavin T-fluorescence-based kinetic assay, infrared spectroscopy, and atomic force microscopy (AFM). Since the valine and glutamate side chains within this segment are strongly involved in the interactions with the insulin receptor, we have focused on the possible implications of the Q → P substitution for insulin’s stability and interactions with the receptor. To this end, comparative molecular dynamics (MD) simulations of the Q5P mutant and wild-type insulin were carried out for both free and receptor-bound (site 1) monomers. The results point to a mild destabilization of the mutant vis à vis the wild-type monomer, as well as partial preservation of key contacts in the complex between Q5P insulin and the receptor. We discuss the implications of these findings in the context of the design of aggregation-resistant insulin analogues retaining hormonal activity.

## Introduction

The self-assembly of polypeptide chains into highly ordered fibrillar aggregates with the characteristic “cross-β” X-ray diffraction pattern, so-called amyloid fibrils, is a generic structural transition accessible to unrelated proteins and peptides.^1,2^ The main reason for undertaking research on amyloid fibrils is the established correlation between histopathological presentation of amyloid deposits and various degenerative maladies such as Alzheimer’s disease, Parkinson’s disease, or type II diabetes mellitus.^3^ Whether mature amyloid fibrils or earlier on-pathway oligomers are causatively linked to illness depends on the type of disease. The high thermodynamic stability of amyloid fibrils under physiological conditions (typically exceeding that of the native state of a globular protein^4^) is paralleled by the robust mechanical characteristic,^5^ both of which have been utilized by living organisms in the form of functional amyloids such as the human Pmel17 protein or bacterial curli protein.^6,7^ Insulin’s propensity to aggregate and form fibrils visible in electron micrographs has been known for a long time.^8^ While the protein has often been used as an insightful model in fundamental biophysical studies of mechanisms of amyloidogenesis, insulin aggregation also poses very concrete and practical problems in medicine and pharmacology. For example, it affects insulin-delivery systems used in the treatment of diabetes and shortens the shelf life of insulin-based pharmaceuticals.^9−13^ Insulin aggregates may also form in the patient’s body – subcutaneously, at the sites of repetitive injections.^14−17^ This so-called insulin-derived amyloidoma (or insulin ball) is an iatrogenic condition associated with insulin resistance.^18,19^ Various molecular and physicochemical factors affecting insulin aggregation have become better understood over the recent years,^20−24^ leading to the development of a plethora of strategies aimed at preventing this process both *in vitro* and *in vivo.*(25−32) Some of these approaches involve the conjugation of aggregation-prone insulin with nanoparticles (e.g., ref (33)) or organic polymers^34,35^ or are based on the addition of tertiary compounds (e.g., refs (31,36)). If plausible, then achieving the same result solely through modifications of the amino acid sequence is advantageous for obvious reasons. In fact, manipulations of insulin’s primary structure and its backbone topology have a long history in the context of amyloid research.^37−39^ Previously, we have identified the disulfide-constrained N-terminal fragment of insulin’s A-chain as a very powerful amyloidogenic stretch which, as a free peptide, aggregates in aqueous solutions at “explosive” rates without detectable lag-phases producing fibrils with the infrared and morphological characteristics similar to those of insulin fibrils.^40−42^ Moreover, the segment (named ACC_1–13_) retains its strong amyloidogenic propensity when tethered to various nonamyloidogenic sequences (e.g., ref (42)), as we have demonstrated on a number of intriguing examples including the unique ATP-incorporating amyloid fibrils in which this segment was extended by an oligolysine fragment.^43,44^ The remarkable aggregation potency of ACC_1–13_ has led us to suspect that this fragment may also play an essential role in insulin aggregation.^40−42,45^ Hence, the working hypothesis underpinning the present study was that amino acid sequence manipulations (e.g., involving proline substitution known to adapt poorly to the β-sheet structure^46−48^) in the N-terminal segment of the A-chain could constitute an alternative approach to the design of aggregation-resistant insulins.

## Materials and Methods

### Samples

**Figure 1:**
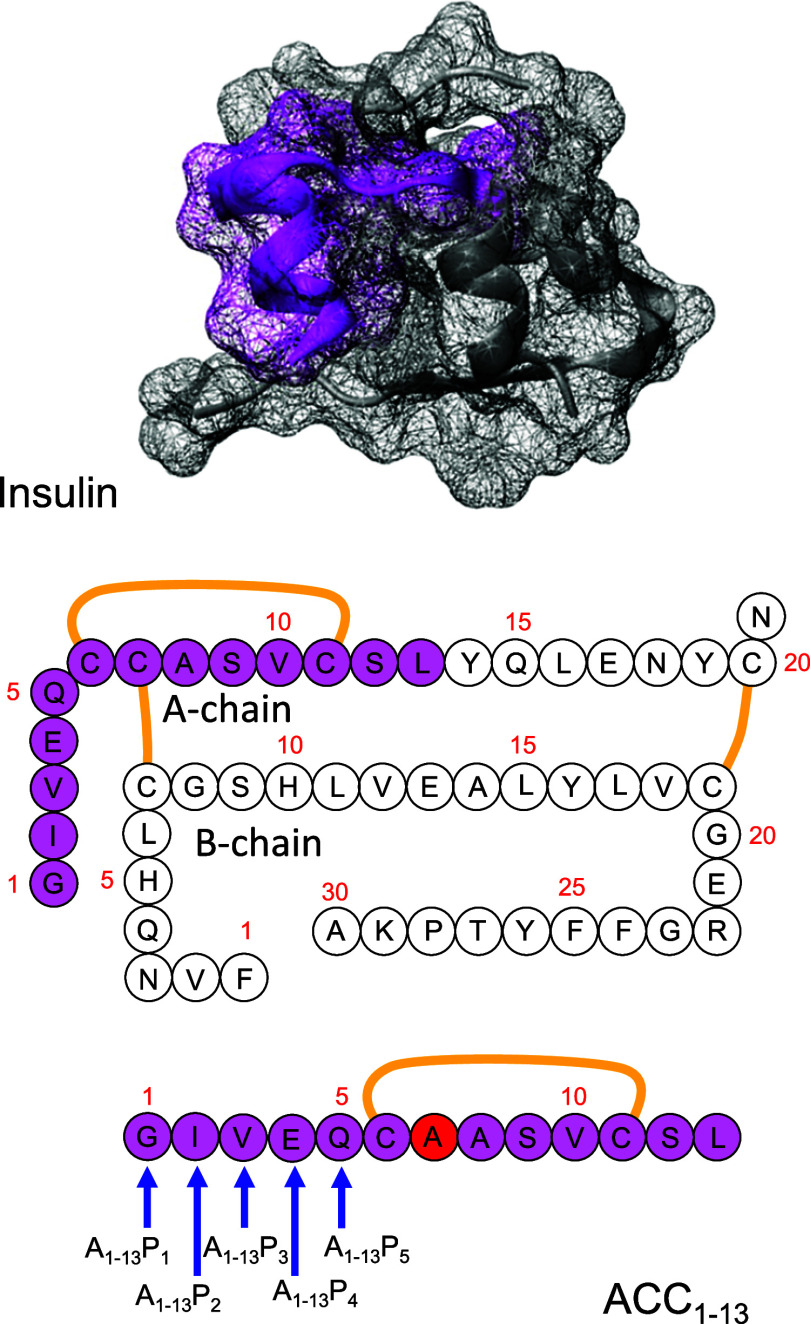
Design of the A_1–13_P*_n_* peptide series. The spatial placement of the N-terminal fragments of insulin’s A-chain (magenta) within the folded insulin monomer (PDB entry: 2a3g). The juxtaposition of amino acid sequences of insulin and A_1–13_P*_n_* peptides; the disulfide bonds are marked in yellow; the sites of substitution with proline within the ACC_1–13_ peptide are marked with blue arrows.

The peptides used in this study were designed by introducing proline substitutions into the pilot sequence (GIVEQ) of the previously characterized highly amyloidogenic N-terminal segment of the insulin A-chain^40−42^ (Figure 1 and Table 1). In all of these peptides (A_1–13_P*_n_*), insulin’s original intrachain Cys6–Cys11 disulfide bond is retained while the native Cys7 residue is substituted with alanine. The peptides, all without N- or C-terminal modifications, were custom-synthesized by Pepscan (currently Biosynth, Lelystad, The Netherlands) typically at a high purity exceeding 95% and delivered as trifluoroacetic acid (TFA) salts. Tris(2-carboxyethyl)phosphine, TCEP, and all other nonpeptidic chemicals were from MilliporeSigma (Sigma-Aldrich). As freeze-dried peptide-TFA salts do not dissolve easily in water, we used the earlier established protocol of gradual solubilization based on sonication-assisted dispersion of peptide-TFA salts in 8 M aqueous guanidine hydrochloride (GdnHCl) solution at a slightly elevated (∼9) pH.^41,42^ The stock peptide solutions (∼3 mg/mL) in concentrated aqueous GdnHCl would remain stable (i.e., without the tendency to aggregate) over a period of several days. However, only freshly prepared stock solutions were used. Peptide aggregation was triggered by the rapid mixing of a portion of the stock peptide solution with a volume of an appropriately acidified aqueous solution of NaCl containing Thioflavin T (ThT). The typical final sample conditions used in the fibrillization protocols were: 0.5 mg/mL peptide dissolved in 1.33 M GdnHCl, 0.05 M NaCl, 20 μM ThT, H_2_O, pH 1.9 or 7.0, as specified. Other details have been described earlier^41,42^ or are placed in figure captions.

**Table 1:** Amino Acid Sequences of Proline-Substituted Insulin-Derived Amyloidogenic Peptides; the Amyloidogenic Propensities According to Cordax and MD Simulation^a^

peptide no.	name (short name)	sequence	Cordax score	β-sheet content
I	ACC_1–13_	GIVEQCAASVCSL	***	0.44
II	A_1–13_P_1_	**P**IVEQCAASVCSL	**	0.43
III	A_1–13_P_2_	G**P**VEQCAASVCSL	*	0.37
IV	A_1–13_P_3_	GI**P**EQCAASVCSL	*	0.30
V	A_1–13_P_4_	GIV**P**QCAASVCSL	*	0.20
VI	A_1–13_P_5_	GIVE**P**CAASVCSL	**	0.22

a Average β-sheet content measured at the end of the MD simulations concluded in Figure S2. Cordax score scale: low (*), intermediate (**), high (***).

### Fibrillization Kinetics (Thioflavin T-Fluorescence Assay)

ThT-fluorescence-based measurements (λ_ex_ = 440 nm/λ_em_ = 485 nm) of peptide fibrillization kinetics were carried out on a CLARIOstar plate reader from BMG LABTECH (Offenburg, Germany) using 96-well black microplates. Typically, each well was filled with a 150 μL portion of freshly prepared peptide solution containing ThT at 20 μM concentration. Measurements were carried out at 37 °C and moderate agitation (300 rpm) for at least 24 h. Each kinetic trajectory was obtained as an average of three independent runs (replicates from the same stock sample solution). Afterward, aggregate samples were collected from the plate and washed with portions of water in order to remove excess salts. Eluted pellets were subjected to atomic force microscopy (AFM) and FT-IR (Fourier transform infrared) spectroscopic measurements.

### Atomic Force Microscopy (AFM)

Aggregate samples collected at the end of the kinetic experiment were washed several times with water. Aqueous suspensions of aggregates were further diluted with water approximately 5 times. A small droplet (10 μL) of such a diluted sample was swiftly deposited onto freshly cleaved mica and left to dry overnight. AFM tapping-mode measurements were carried out using a Nanoscope III AFM from Veeco Instruments (Plainview, NY) and TAP300-Al sensors (res. frequency 300 kHz) from BudgetSensors (Sofia, Bulgaria). Cross-sections of selected specimens superimposed on the amplitude images were obtained from the corresponding height data.

### Attenuated Total Reflectance (ATR) FT-IR Measurements

Samples of aggregated peptides formed in the course of the kinetic experiments were centrifuged and washed several times with equal portions of water in order to remove excess salts (vibrational bands of GdnHCl and TFA would overlap with the amide I band of insulin). Salt-depleted suspensions of fibrils were deposited on the diamond surface of a single-reflection diamond ATR accessory of a Nicolet iS50 FT-IR spectrometer from Thermo Fisher Scientific (Waltham, MA) equipped with a DTGS detector. The samples were dried *in situ* within a few minutes using a stream of dry air. Typically, for a single ATR-FT-IR spectrum, 32 interferograms of 2 cm^–1^ nominal resolution were coadded. Due to the difficulty of determination of real values of refractive indexes of amyloid aggregates, only uncorrected ATR-FT-IR data are shown. Spectral data processing was limited to subtracting the water vapor spectrum using GRAMS software (Thermo Fisher Scientific).

### Molecular Dynamics (MD) Simulations

#### MD-Based Comparative Analysis of the Mutant of ACC_1–13_ Assemblies and Insulin Monomers

Molecular dynamics (MD) simulations were conducted using the AMBER CUDA^49,50^ and the FF14SB force field. The initial structure of the ACC_1–13_ assembly consisting of six monomers was obtained thanks to the courtesy of Dr. Kolinski.^51^ Proline substitutions were introduced using Yasara View.^52^ In all ACC_1–13_ monomer chains, side chains of the glutamate residues were protonated. Each initial system was solvated with TIP3P water molecules and neutralized with Na^+^ and Cl^–^ ions within a rectangular box, ensuring a minimum distance of 16 Å between any protein atom and the box boundary. The resulting simulation boxes consisted of 9056 solvent molecules for G1P, 8755 for I2P, 8929 for V3P, 8934 for E4P, 8742 for Q5P, and 8928 H_2_O molecules for the wild-type (WT) ACC_1–13_ assembly. For the solvated systems of insulin monomers, the WT monomer system contained 8113 water molecules, and the Q5P mutant contained 8285 water molecules. The preliminary equilibration process involved multiple stages. Initially, the system was optimized using 800 steps of the steepest descent algorithm followed by 200 steps of the conjugate gradient method. Subsequently, the systems were gradually heated to 300 K over the course of the first 5 ns with backbone atoms constrained in the NPT ensemble. This was followed by a 5 ns-long equilibration phase during which only C_α_ atoms were constrained. For subsequent production runs, no constraints were imposed on heavy atoms. Bond lengths involving hydrogen atoms were maintained using the SHAKE algorithm,^53^ allowing the integration time step of 2 fs. The simulations were performed by using the GPU-accelerated pmemd code with a real-space cutoff of 8.0 Å and Langevin dynamics with a collision frequency of 2 ps^–1^. For each proline substitution in ACC_1–13_, 10 independent runs, each lasting 100 ns, were carried out. Likewise, for insulin monomers, both for the WT and the Q5P mutant, we conducted 10 independent simulations, each lasting 500 ns.

In order to perform cluster analysis, 100 snapshots from the final stages of simulations for each type of insulin (WT/Q5P) have been collected. In the next step, these structures were organized into five clusters by using the K-means algorithm. Subsequently, centroids from the largest clusters were identified for each insulin type using Clusco software.^54^

#### MD Analysis of Insulin Monomer (WT/Q5P)–Insulin Receptor Interactions

The initial structures for the MD simulations involving the insulin receptor bound by four insulin molecules were obtained from the Protein Data Bank (PDB) entry 6sof.^55^ In cases where fragments were absent from the 6sof structure, the missing portions were reconstructed using Modeller software.^56^ The resulting model was then neutralized using 43 Na^+^ and 5 Cl^–^ ions. Subsequently, the system was solvated with TIP3P water molecules within a rectangular simulation box. The minimum distance between any protein atom and the boundaries of the box was maintained at 24 Å, resulting in a system consisting of 364,074 atoms and 110,646 (WT) or 110,650 (Q5P) water molecules. The equilibration process encompassed several stages. Initially, each system underwent energy minimization of 2000 steps of the steepest descent algorithm followed by an additional 2000 steps using the conjugate gradient method. Subsequently, gradual heating was carried out, raising the temperature to 300 K over a period of 5 ns. During this phase, the backbone atoms were restrained to their initial positions within the NPT ensemble. This was followed by a 25 ns-long equilibration in the NVT ensemble. In the final 25 ns-long equilibration stage, all C_α_ atoms were constrained except for the regions that were reconstructed using Modeller, which were left unconstrained. In order to enhance the computational efficiency, hydrogen mass repartitioning was applied, and an integration time step of 4 fs was employed.^57^ Finally, production simulations were conducted. For each system, a total of five independent simulations were performed, each 500 ns-long.

We have defined a contact between two residues when any atom from one residue is present for at least 50% of the analyzed time within a distance of 6 Å from atoms of the other residue. Only the second half of the simulations (250 ns) is considered in this analysis.

## Results and Discussion

ACC_1–13_, the N-terminal disulfide-constrained stretch of the A-chain (GIVEQCAASVCSL), retains the α-helical conformation as long as it remains an integral part of the folded insulin monomer. However, ACC_1–13_ reveals its extremely amyloidogenic character when separated from the parent protein.^42^

We have shown earlier that ACC_1–13_ is capable of enforcing a cooperative amyloidogenic behavior when coupled with various nonamyloidogenic peptide fragments.^42−45,58−60^ This holds true, in particular, for the highly aggregation-prone “H-fragment” released upon partial proteolysis of insulin with pepsin.^40^ Our initial motivation here was to examine to what extent β-sheet-breaking proline substitutions within the initial GIVEQ part of ACC_1–13_ peptide could attenuate its extreme amyloidogenic potency.

**Figure 2:**
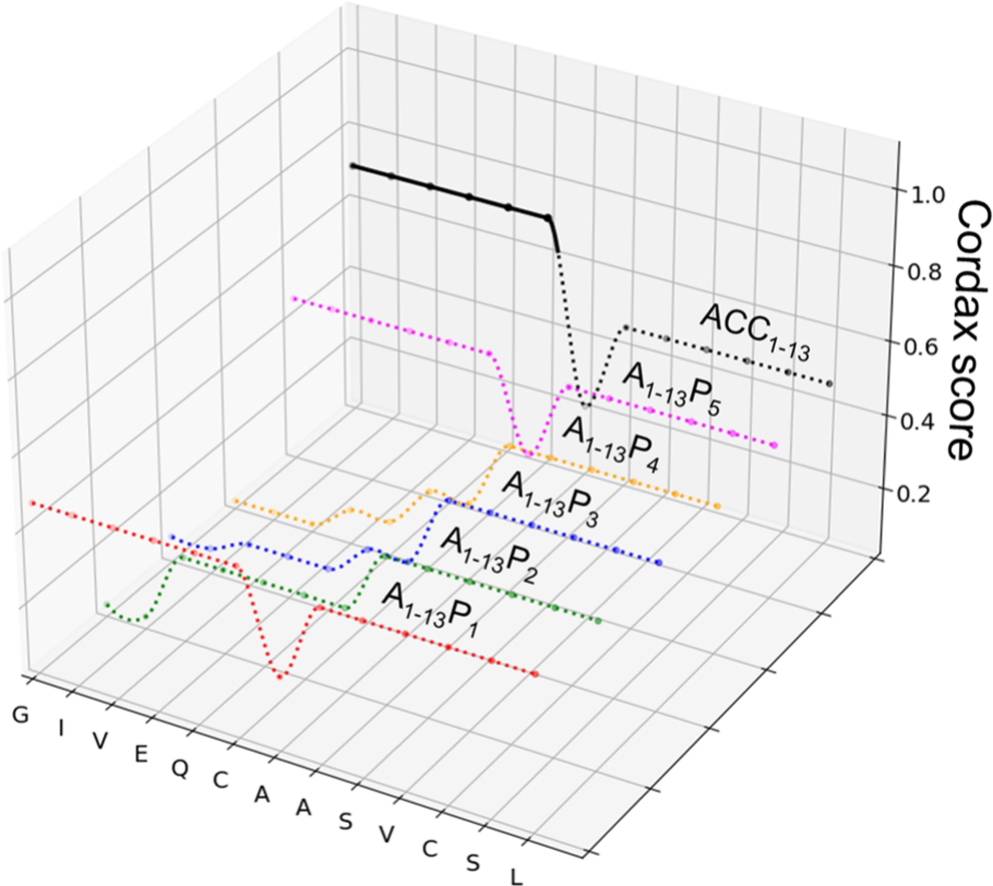
Cordax-based prediction of amyloidogenic propensities of A_1–13_P*_n_* peptides; the lines plotted to guide the eye become solid at sites where the amyloidogenic propensity is significant. Among the sequences examined here, only ACC_1–13_ (WT) exceeds the threshold of amyloidogenicity (0.61 score on the Cordax scale) depicted by the thickened line, whereas proline substitutions at positions 2, 3, and 4 reduce it substantially. A 2D representation of the data is given in Figure S1.

The proline substitution sites within ACC_1–13_ are presented in Figure 1 and Table 1. Since certain computational tools such as TANGO and WALTZ developed to predict the amyloidogenic potential of peptides performed rather poorly in the case of ACC_1–13,_^42^ here we have employed Cordax—a structure-based machine learning approach exploring sequence determinants of the amyloid-forming potential.^61^ This was done to carry out a preliminary assessment of the amyloidogenicity of ACC_1–13_ and its proline-modified analogues. The results presented in Figure 2 turned out to be quite encouraging: the Cordax algorithm correctly predicted the already experimentally confirmed aggregation propensities of ACC_1–13_ (see Figure S1 for a 2D representation of the Cordax data).

**Figure 3:**
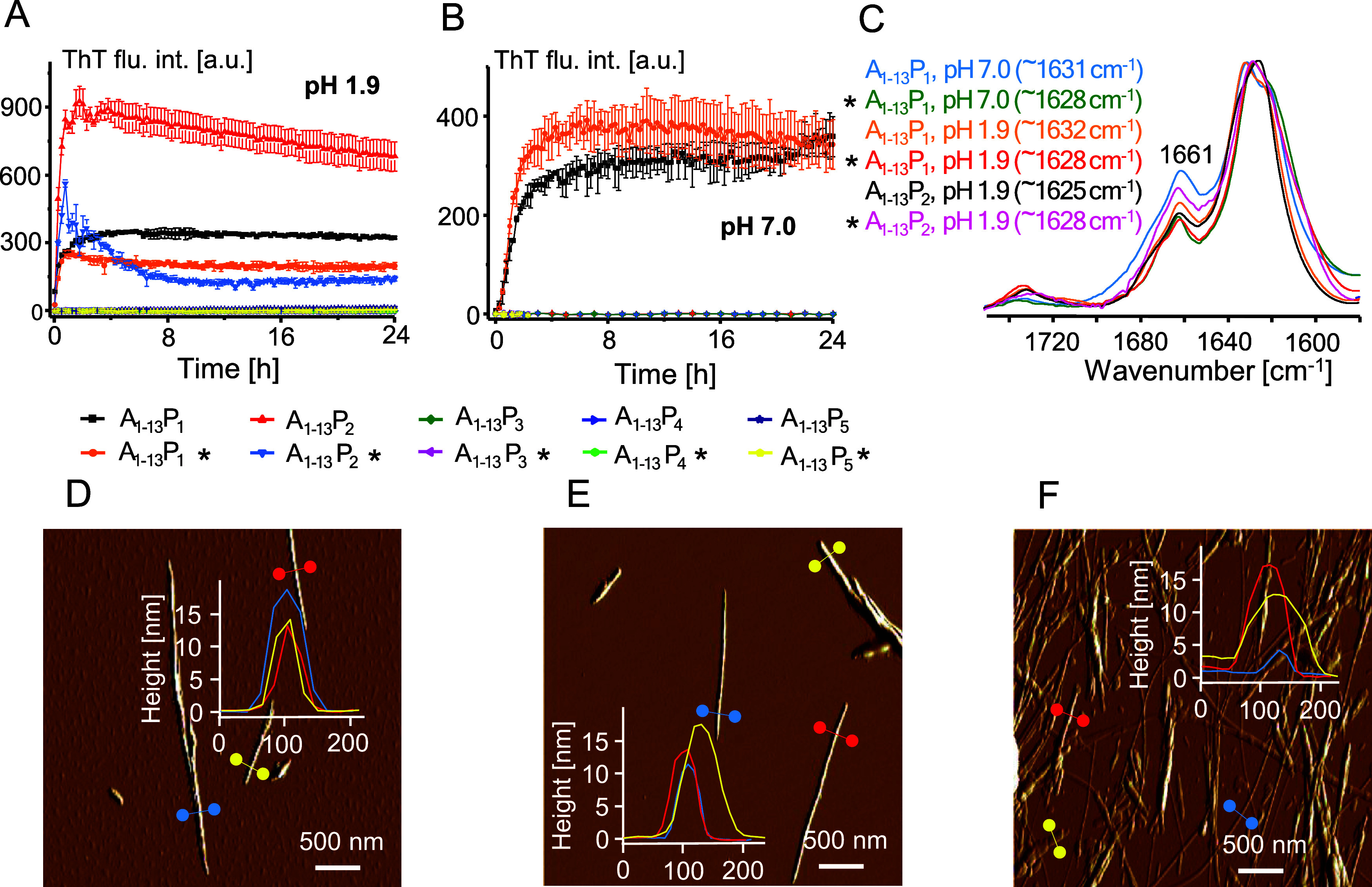
Experimental verification of the amyloidogenic propensities of A_1–13_P*_n_* peptides. ThT-fluorescence-intensity-based kinetic trajectories of peptide fibrillization at 37 °C and low (A) and neutral (B) pH and in the absence and presence of the disulfide-reducing agent (conditions: 0.5 mg/mL peptide dissolved in 1.33 M GdnHCl, 0.05 M NaCl, 20 μM ThT, H_2_O, pH adjusted as indicated; the disulfide-reducing conditions were maintained in the selected samples by the presence of TCEP added at the concentration of 1.7 mg/mL; samples corresponding to the reducing conditions are marked with “*”). ATR-FT-IR spectra of dried aggregates of A_1–13_P_1_ and A_1–13_P_2_ formed under different conditions, as indicated (C); the amide I band maxima are given in parentheses. Amplitude AFM images of aggregated A_1–13_P_1_, pH 1.9 (D), A_1–13_P_1_, pH 1.9 + TCEP (E); A_1–13_P_2_, pH 1.9 (F); overlaid are cross-sections of selected specimens.

The predicted impact of proline substitutions is rather intuitive according to the Cordax data: the residue-resolved amyloidogenic propensity decreases in the vicinity of the Pro residue. We note that proline substitutions at sites 1 (glycine) and 5 (glutamine) appear to have similar moderate aggregation-mitigating effects, while the consequences of the substitutions at positions 2, 3, and 4 are much more pronounced. Recently, we utilized multiscale molecular modeling methods for the structure predictions of the short fibrillar aggregates, including the ACC_1–13_ model.^51,62^ Consequently, we conducted molecular dynamics simulations for these assemblies, including those with proline substitutions. The results of these simulations are presented in Figure S2 and Table 1. The most stable forms of ACC_1–13_, as determined by the average β sheet content and structural stability, are the wild type and the peptide A_1–13_P_1_. Mutations at positions 2, 3, 4, and 5 destabilize the ACC_1–13_ amyloid structure, with positions 4 and 5 exhibiting the strongest disruption of the β sheet structure. The actual amyloidogenic tendencies of A_1–13_P*_n_* amino acid sequences were verified experimentally using custom-synthesized peptides, ThT-fluorescence assay, and infrared spectroscopy (Figure 3). At the start of each kinetic experiment, a volume of solution of a given peptide in concentrated GdnHCl was rapidly diluted with an excess of pH-adjusted NaCl solution so that the denaturant concentration would drop below the level preventing instant self-association of aggregation-competent peptides (Materials and Methods). The aggregation screening test was carried out in both acidic (pH 1.9) and neutral environments and in the presence and absence of a fast-acting disulfide-reducing agent (TCEP). It is important to stress that of the four different sets of environmental conditions (low/neutral pH, reducing/nonreducing conditions), the pH = 7.0/no TCEP case is the one with actual physiological relevance for insulin aggregation. The impact of the other three sets of environmental conditions was analyzed out of mechanistic interests since insulin aggregation is usually studied at low pH, while the Cys6–Cys11 disulfide bond has been shown to enhance aggregation of several synthetic peptides derived from the N-termini of insulin’s A-chain.^41^

The kinetic data in panel A of Figure 3 depict very fast self-assembly of ThT-positive amyloid fibrils at the low pH only in the case of A_1–13_P_1_ and A_1–13_P_2_ peptides.

The data obtained at the neutral pH (Figure 3B) show that A_1–13_P_2_ loses its amyloidogenicity, most likely due to the Glu residue at position 4 becoming ionized. Repulsive Coulombic interactions between closely arranged Glu side chains could constitute an additional destabilizing factor (apart from the proline substitution) in an in-register stack of parallel β-strands in which spatial separation of side chains of identical residues is restricted. Importantly, in the absence of proline substitutions, the “wild type” ACC_1–13_ (‘A’) peptide readily forms fibrils at neutral pH (Figure S3^45^). Thus, the absence of aggregation of A_1–13_P_2_ at pH 7 clearly arises from an interplay of electrostatics and the β-sheet-breaking effect of proline. We note that the reduction of disulfide bonds appears to have little effect on the aggregation behavior of these peptides. One could speculate that the removal (through the TCEP-induced reduction) of the disulfide-induced conformational constraints may help accommodate the structural tensions caused by the proline presence. Hypothetically, this could easily offset the loop entropy effect associated with the intact disulfide bond which had been argued to contribute to the amyloidogenic propensity of similar peptides.^41^

The formation of ThT-positive aggregates in the kinetic experiments has been correlated with the presence of β-sheet structure, as reflected by the infrared spectra of the aggregates shown in Figure 3C. Regardless of the physicochemical conditions of aggregation, the frequency of the main spectral component of the amide I band of precipitates of A_1–13_P_1_ and A_1–13_P_2_ collected at the end of the kinetic experiment is in the range between 1625–1632 cm^–1^ which, in the absence of an exciton-split high-frequency component above 1680 cm^–1^ (as is the case here), is indicative of the presence of the parallel β-sheet structure typically observed for insulin amyloid fibrils.^40^ Aggregated A_1–13_P_1_ and A_1–13_P_2_ share similarities with insulin amyloid also on the morphological level as revealed by the AFM images shown in Figure 3D–F. The most common straight unbranched specimens are rather thick, typically 12–15 nm in diameter, which indicates lateral alignment of 3–4 protofilaments.

**Figure 4:**
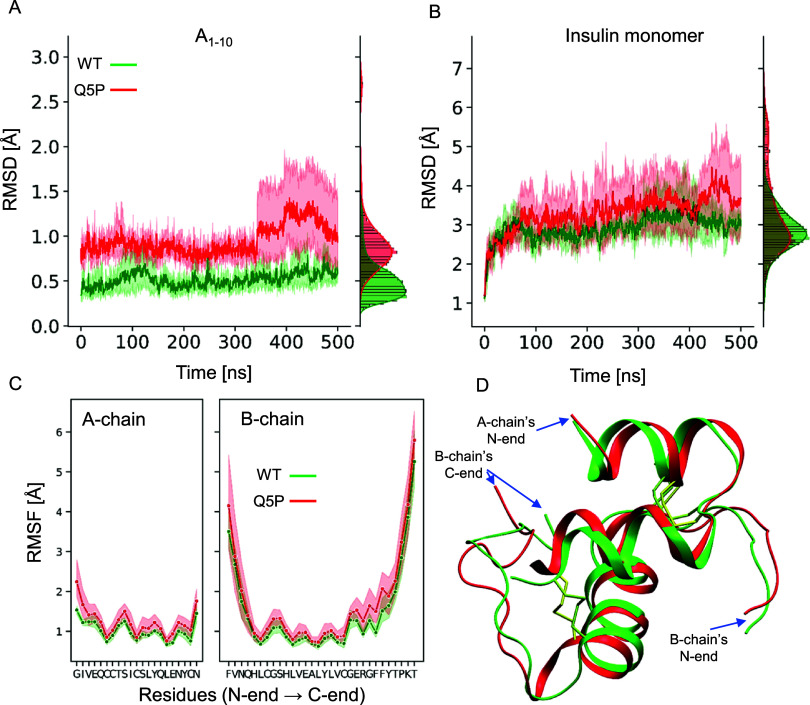
*In silico* comparison of the conformational stabilities of the WT human insulin monomer and the proline-substituted Q5P mutant. Changes of RMSD of the backbone Cα atoms within the N-terminal segment of the A-chain (first 10 residues, A) and the whole insulin monomer (B) during 500 ns-long all-atom MD simulations (averaged over three independent runs); the histograms on the right side depict time-averaged spreads of RMSD. (C) Corresponding time-averaged RMSF values for each residue of the A- and B-chain. (D) Superimposition of snapshots of insulin conformations (WT, green; Q5P variant, red) at the end of the 500 ns-long simulations.

The amyloidogenicity of ACC_1–13_ is not fully understood. We have shown earlier that the dissection of this peptide into shorter fragments, ACC_1–5_ (GIVEQ) and ACC_6–13_, results in the complete loss of the amyloidogenic potency (ref (45); see also Figure S3). The juxtaposition of the Cordax-based predictions with the experimental data presents an excellent agreement as far as the impact of proline substitutions at positions 3 and 4 is concerned. On the other hand, the computational approach clearly overestimates the impact of these mutations at position 2 while underestimating it at position 5. To the extent that the amyloidogenic behavior of ACC_1–13_ approximates the aggregation propensity of the whole insulin monomer, the outcome of the proline scan presented so far may provide important clues for the design of aggregation-resistant insulin. The GIVEQ region is generally conserved among insulin types from various organisms, as some of these residues are involved in the docking interactions with the receptor.^63−66^ Proline mutations at positions 1 (Gly) and 2 (Ile) yield an aggregation propensity similar to that of the wild-type ACC_1–13_. Positions 3 (Ile), 4 (Glu), and 5 (Gln) in the A-chain of insulin are crucial for forming a surface that binds and interacts with the insulin receptor.^64,65^ However, the substitution of position 5 (Glu), e.g., with alanine, only slightly reduces the potency of such modified insulin.^66^ Hence, for a consideration of a hypothetical insulin mutant that could be resistant to aggregation due to proline substitution in the pilot part of the ACC_1–13_ segment, we have selected a proline mutant at position 5 (Q5P) to assess *in silico* its plausible stability and receptor-binding properties. We have carried out MD simulations of the Q5P insulin monomer at 300 K and compared them with those conducted for the nonmutated monomer. In Figure 4A,B, RMSD trajectories of both types of monomers are juxtaposed. The increased level of fluctuations in the mutant is even more pronounced within the A_1–10_ region of insulin compared to that of the whole monomer. The PCA analysis of the data further highlights the impact that the Q5P mutation has on the overall dynamics of the monomer (Figure S4). The outcome of the calculation of individual residue flexibilities expressed as root-mean-square fluctuation, RMSF, is plotted for A- and B-chains in Figure 4C.

**Figure 5:**
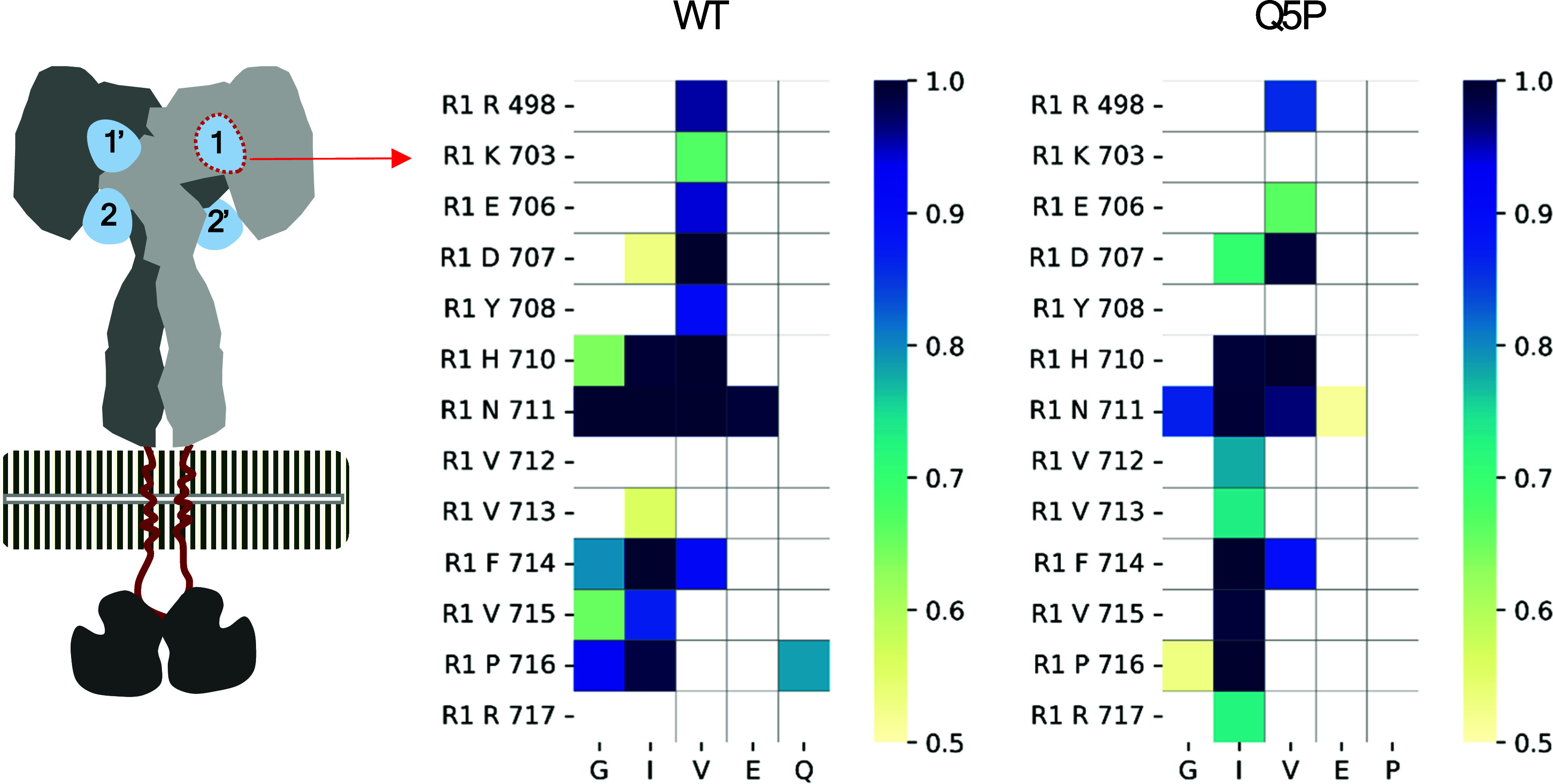
Contact maps of insulin–insulin receptor (IR) interactions at the receptor’s site “1” with the A-chain N-terminal region (5 residues) obtained for monomers of WT insulin and the Q5P mutant. The color scale reflects the contact frequency. For the sake of clarity, only contacts with a frequency exceeding 50% of the time are marked. Residues involved in insulin binding (horizontal sequence, 1 letter code) at receptor site 1 (vertical sequence) that are present in the WT (left) are also conserved in the mutant (right), except for Gly 1 and Phe 714.

The RMSF levels of the Q5P mutant are consistently elevated throughout the primary structure of both chains, although the GIVEP stretch is clearly one of the regions more affected by the mutation. The fact that the direct vicinity of the mutation site is more affected in terms of conformational dynamics than the more remote parts of the monomer is somehow intuitive. It should be stressed, however, that the overall structure of the Q5P mutant remains very similar to the WT monomer. In Figure 4D, snapshots of insulin conformations of WT and Q5P variants obtained after the 500 ns-long simulations are superimposed. According to the cluster analysis carried out on these conformations (Materials and Methods), both monomers are quite similar, which is reflected by the relative RMSD of 1.6 Å between centroids of the largest clusters. Hence, based on the MD data, the overall impact of the Q5P mutation could be moderate, allowing the monomer to retain most of its native folding. This result has become a starting point for the preliminary analysis of possible consequences of this mutation on the interactions with the insulin receptor. Since only one insulin binds to the receptor with high affinity and site 2 weakly interacts with the GIVEQ sequence,^65^ the scope of our analysis has been limited to the possible interactions of insulin monomers (Q5P vs WT) with the receptor at site 1. The key result of these computations (see Materials and Methods) is shown in Figure 5, presenting contact maps of insulin–insulin receptor (IR) interactions at the receptor’s site “1” narrowed to the 5-residue-long A-chain’s N-terminal region for the nonmutated insulin monomer (WT) and the Q5P mutant. The complete contact maps for the whole insulin monomer are shown in Figure S5.

Predictably, the substitution has some consequences for insulin-receptor interactions. While the glutamine side chain itself contributes marginally to the binding (weak interactions with Pro 716 of the receptor), its substitution with proline affects the patterns of interactions of all of the four preceding side chains of glycine, isoleucine, valine, and glutamate within the insulin segment. For example, contacts of glycine with residues Phe 714 and Pro 716 are weakened, as are the contacts of valine with residues Glu 706 and Tyr 708 or of glutamate with the residue Asn 711. There are a few biochemically predicted interactions involving the GIVEQ segment of insulin’s A-chain and the residues at site 1 of the receptor, namely, Gly A1–Phe 714, Gly A1–Asn 711, Ile A2–Phe 714, Val A3–His 710 or Val A3, and Asn 711.^63−65^ The only crucial interaction lost due to the mutation is the Gly A1–Phe 714 pair (Figure 5). The overall pattern of affinity does, however, appear to be maintained. Given the level of prediction accuracy that one may expect from such an entirely *in silico* approach, this result clearly requires experimental verification. It should be stressed, however, that the simulations carried out here do not point to any fundamental steric conflict or repulsive interactions that would rule out the possibility that the Q5P mutant could, indeed, retain the hormonal activity of insulin. A superimposition of WT and Q5P insulin monomers after the receptor docking is presented in Figure S6.

## Conclusions

In conclusion, we have shown that certain proline substitutions within the pilot GIVEQ segment of ACC_1–13_, the strongly amyloidogenic N-terminal fragment of insulin’s A-chain, switch off the propensity to aggregate entirely at both acidic and neutral pH. In light of the fact that ACC_1–13_ is likely to contribute strongly to the aggregation propensity of insulin, we argue that proline substitutions in this part of the parent protein could guide the design of an aggregation-resistant hormone. As biosynthesis of such proline-substituted insulin analogues is a complicated and costly enterprise, here we have attempted to assess the conformational stability and plausible interactions patterns with the receptor of a selected proline-substituted mutant (Q5P) using MD simulations. The results suggest that such a mutant would maintain the key structural characteristic of native insulin and that the amino acid substitution does not create a major steric conflict or pattern of repulsive interactions that would rule out binding to the insulin receptor at site 1. Hence, proline substitutions within the N-terminal segment of the A-chain could complement the various approaches used in the design of stable insulin refractive to aggregation.

## Data Availability

The data are available from the authors on reasonable request.

## Supporting Information Available

Additional MD data on the stability of A_1–13_P*_n_* assemblies; experimental data on amyloidogenic properties of ACC_1–5_, ACC_6–13_, and unsubstituted ACC_1–13_ fragments; PCA analysis of WT and Q5P monomers; and large contact maps of insulin–insulin receptor (IR) interactions for WT and Q5P insulin monomers (PDF)
