# Supplementary material for: Clues to the Design of Aggregation-Resistant Insulin from Proline Scanning of Highly Amyloidogenic Peptides Derived from the N-Terminal Segment of the A-Chain
Source: Mol Pharm. 2024 Mar 25;21(4):2025–33. doi: 10.1021/acs.molpharmaceut.4c00077 (PMC10988558; doi:10.1021/acs.molpharmaceut.4c00077)
Supplement: Supplementary file 1 — mp4c00077_si_001.pdf [file mp4c00077_si_001.pdf]

SUPPORTING INFORMATION

for

Clues to the design of aggregation-resistant insulin from proline scanning of highly amyloidogenic peptides derived from the N-terminal segment of the A-chain

by

Wojciech Puławski, Robert Dec, and Wojciech Dzwolak

Contents:

1. Cordax results obtained for A_{1-13}-P_n peptides: a 2D version of Figure 2.

2. MD-based comparative analysis of A_{1-13}-P_n assemblies.

3. Amyloidogenic properties of ACC_{1-5}, ACC_{6-13} and ACC_{1-13} fragments: ThT fluorescence assay, IR spectra, AFM images of selected fibrillar specimens.

4. PCA of WT and Q5P insulin monomers.

5. Large contact maps of insulin-insulin receptor (IR) interactions at the receptor's site '1' for non-mutated insulin monomer (WT) and the Q5P mutant.

6. Superimposition of snapshots of insulin monomers after the docking with Insulin Receptor (at site 1).

1. Cordax results obtained for A1-13-Pn peptides: a 2D version of Figure 2.

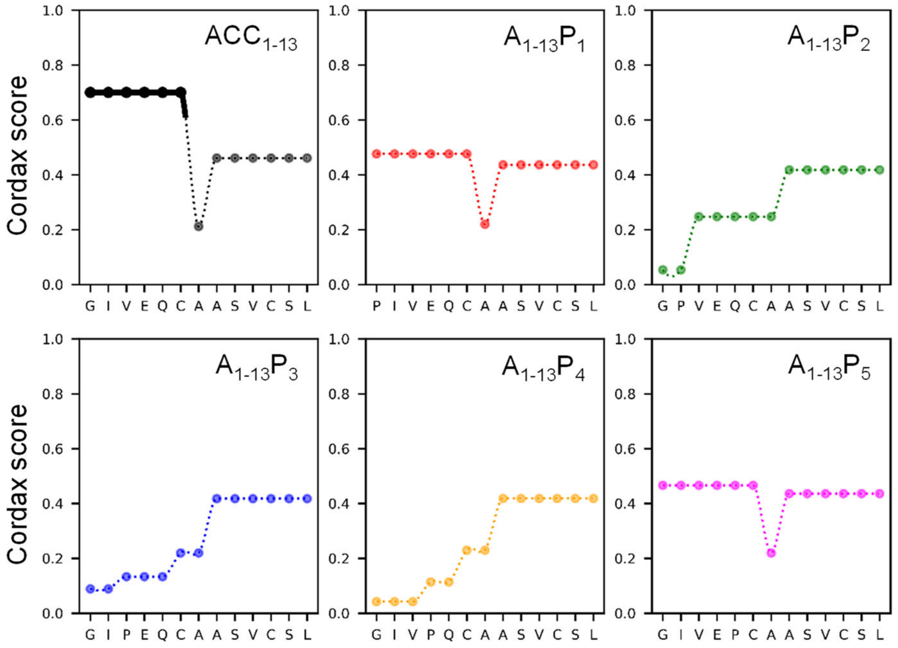

Figure S1. Cordax-based prediction of amyloidogenic propensities of $A_{1\text{-}13}P_n$ peptides – a 2D representation of the data shown in Figure 2 of the main article; the lines plotted to guide the eye become solid at sites where the amyloidogenic propensity is significant. Among the sequences examined here, only $ACC_{1\text{-}13}$ (WT) exceeds the threshold of amyloidogenicity (0.61 score in Cordax scale) depicted by the thickened line, whereas proline substitutions at positions 2, 3, and 4 reduce it substantially.

2. MD-based comparative analysis of A1-13-Pn assemblies.

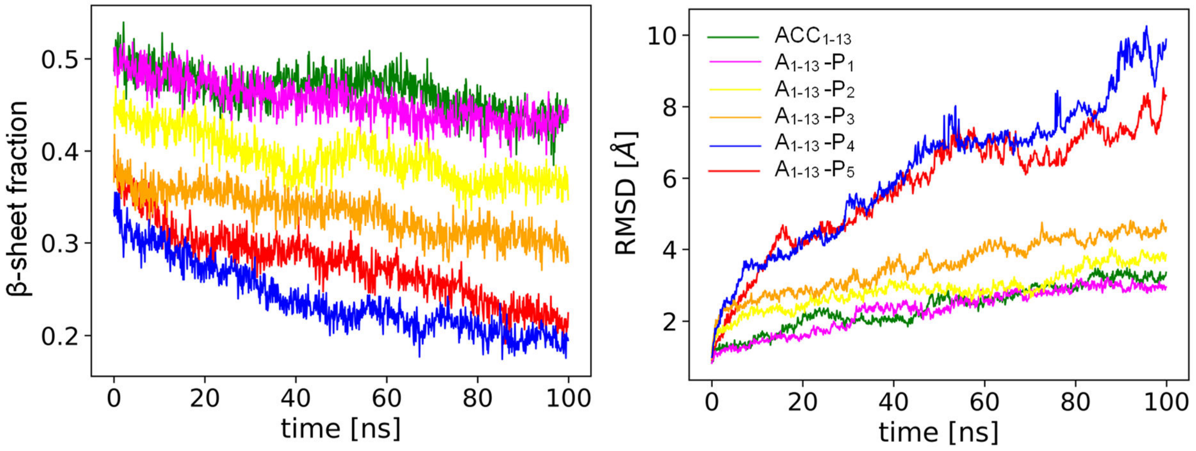

Figure S2. MD-based comparative analysis of $A_{1\text{-}13}\text{-}P_n$ assemblies. Comparison of average β-sheet content (left) and stability of amyloidal stacks of the $A_{1\text{-}13}\text{-}P_n$ peptide chains (right). The β-sheet content is defined as a ratio of the number of the C_α carbons involved in the β-sheet

structure (as recognized by the Stride algorithm [S1]) to all of the Cα carbons in the molecule, whereas stability is measured by backbone Cα root-mean-squared deviation from initial structure. The wild-type peptide and A_{1-13}-P_1 show the highest stability among the variants. Mutations at positions 2, 3, 4 and 5 result in destabilization of the ACC_{1-13} amyloid structure, with positions 4 and 5 displaying significantly reduced amyloidogenicity.

3. Amyloidogenic properties of ACC_{1-5}, ACC_{6-13} and ACC_{1-13} fragments: ThT fluorescence assay, IR spectra, AFM images of selected fibrillar specimens.

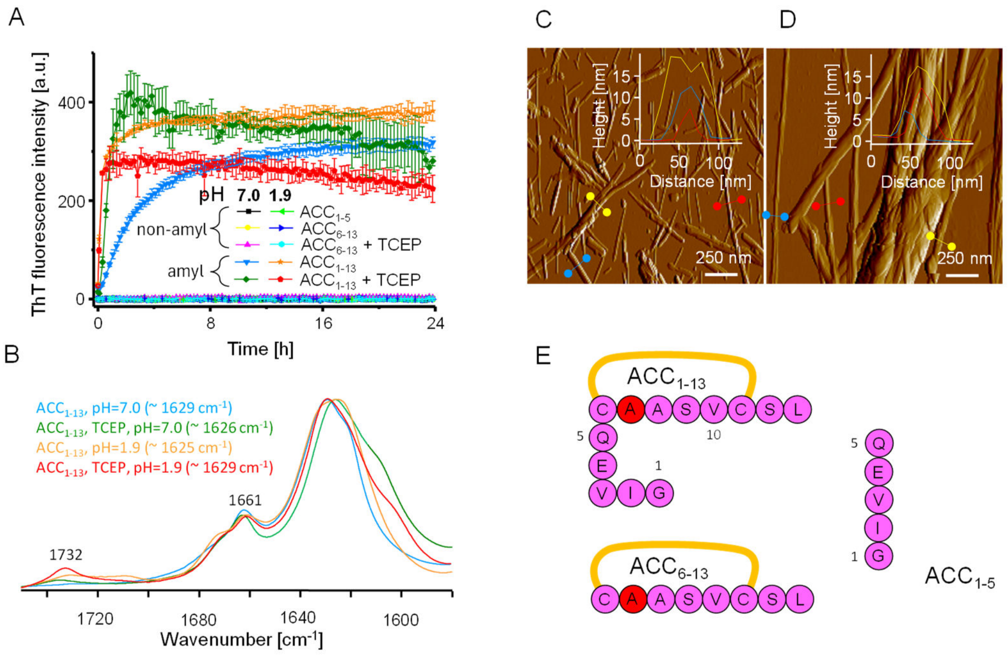

Figure S3. Aggregation of the whole ACC_{1-13} peptide and its ACC_{1-5} and ACC_{6-13} fragments at neutral and acidic pH and in the presence and absence of disulfide-reducing agent (TCEP) at 37 °C. (A) Kinetic trajectories of fibrillization according to the ThT fluorescence intensity (conditions: 0.5 mg/mL peptide dissolved in 1.33 M GdnHCl, 0.05 M NaCl, 20 μM ThT, H_2O, pH adjusted as indicated; the disulfide-reducing conditions were maintained in the selected samples by the presence of TCEP added at the concentration of 1.7 mg/mL). (B) ATR-FT-IR spectra of eluted with excess of water and subsequently dried aggregated samples collected at the end of the kinetic experiments; the amide I band maxima are given in parenthesis. AFM amplitude images of ACC_{1-13} aggregated at pH 1.9 in the absence (C) and presence (D) of TCEP. (E) Amino acid sequences of the examined peptides.

4. PCA of WT and Q5P insulin monomers.

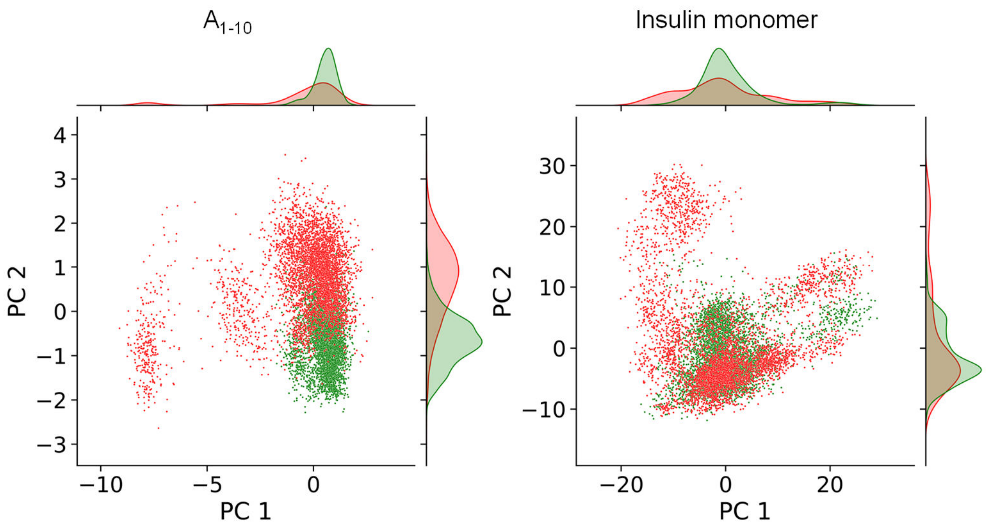

Figure S4. Principal Component Analysis (PCA) of WT and Q5P mutant insulin. The plots illustrate projections of the two dominant modes (PC1 and PC2) for WT (green) and Q5P insulin mutant (red) based on MD simulations.

The analysis for residues 1 to 10 of the A-chain is presented on the left, while that for the whole amino acid sequence of insulin monomer (residues 1 to 51) is on the right. Each point corresponds to a mode value for an individual frame in the trajectory. Histograms of PC1 reveal a similar peak for both WT and mutant insulin, but the mutant exhibits a broader distribution. Notably, PC2 displays distinct distributions for residues 1-10, indicating different dynamics between the two cases, consistent with RMSD plots.

Methods: Principal Component Analysis (PCA) was performed using CPPTRAJ/Ambertools 22 [S2, S3]. The initial half of all simulations was discarded and only the latter half was used for analysis. Subsequently, the remaining frames were subjected to alignment to eliminate translational and rotational motions. An average structure was then generated, and the coordinate covariance matrix was computed. The eigenvectors were obtained through matrix diagonalization. Finally, the coordinates from each trajectory were projected onto the two primary eigenvectors to derive the projection values corresponding to the identified modes.

5. Full contact maps of insulin-insulin receptor (IR) interactions at the receptor's site '1' for non-mutated insulin monomer (WT) and the Q5P mutant.

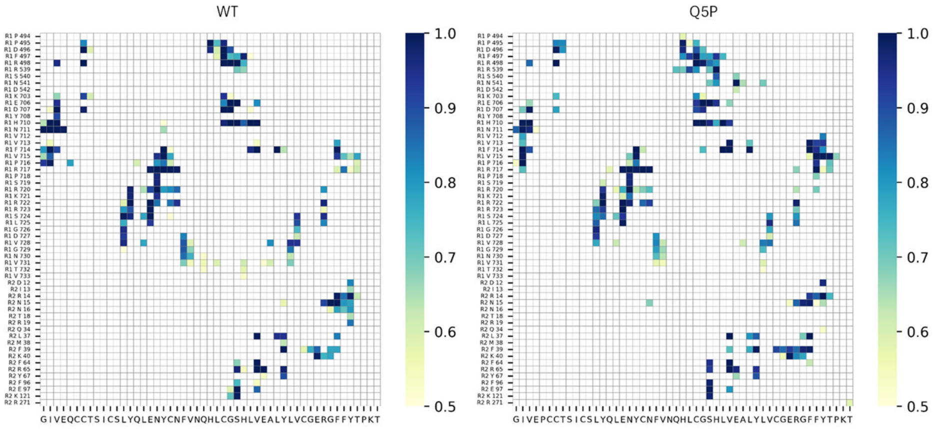

Figure S5. Contact maps of insulin-insulin receptor (IR) interactions at the receptor's site '1' obtained for non-mutated insulin monomer (WT) and the Q5P mutant (affinities across the whole amino acid sequence of insulin). The color scale reflects contact frequency. For the sake of clarity, only contacts with the frequency exceeding 50 % of time are marked. R1, R2 denote first or second monomer of the receptor.

6. Superimposition of snapshots of insulin monomers after the docking with Insulin Receptor (at site 1).

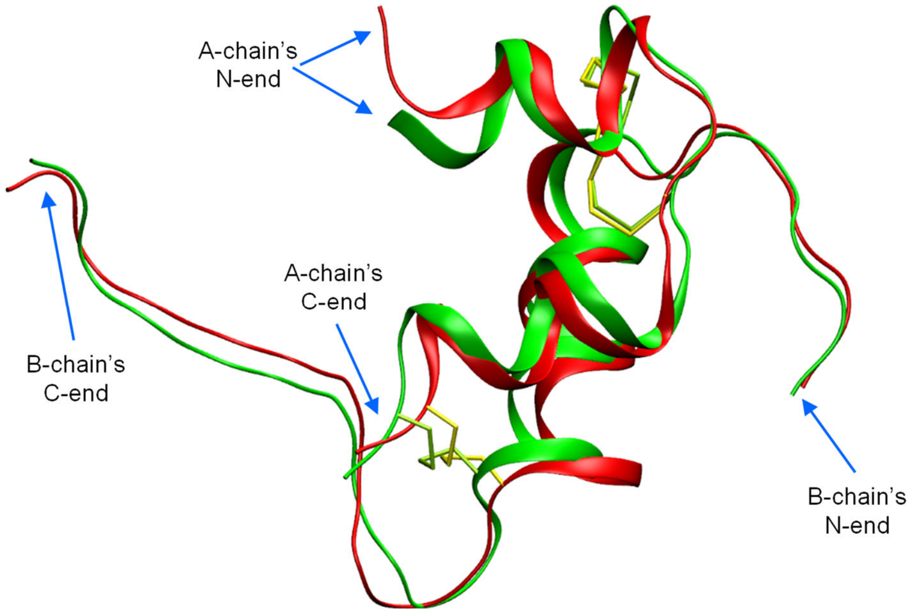

Figure S6. Superimposition of the largest clusters of insulin conformations (WT – green, Q5P variant – red) after the docking with Insulin Receptor (site 1) at the end of the 500 ns-long simulations. Calculated RMSD of Cα atom positions is 1.36 Å.

References:

[S1] Frishman, Dmitrij, and Patrick Argos. "Knowledge-based protein secondary structure assignment." *Proteins: Structure, Function, and Bioinformatics* 23.4 (1995): 566-579.

[S2] Roe, D. R., & Cheatham III, T. E. (2013). PTRAJ and CPPTRAJ: software for processing and analysis of molecular dynamics trajectory data. Journal of chemical theory and computation, 9(7), 3084-3095.

[S3] AmberTools23 D.A. Case, H.M. Aktulga, K. Belfon, I.Y. Ben-Shalom, J.T. Berryman, S.R. Brozell, D.S. Cerutti, T.E. Cheatham, III, G.A. Cisneros, V.W.D. Cruzeiro, T.A. Darden, N. Forouzesh, G. Giambaşu, T. Giese, M.K. Gilson, H. Gohlke, A.W. Goetz, J. Harris, S. Izadi, S.A. Izmailov, K. Kasavajhala, M.C. Kaymak, E. King, A. Kovalenko, T. Kurtzman, T.S. Lee, P. Li, C. Lin, J. Liu, T. Luchko, R. Luo, M. Machado, V. Man, M. Manathunga, K.M. Merz, Y. Miao, O. Mikhailovskii, G. Monard, H. Nguyen, K.A. O'Hearn, A. Onufriev, F. Pan, S. Pantano, R. Qi, A. Rahnamoun, D.R. Roe, A. Roitberg, C. Sagui, S. Schott-Verdugo, A. Shajan, J. Shen, C.L. Simmerling, N.R. Skrynnikov, J. Smith, J. Swails, R.C. Walker, J. Wang, J. Wang, H. Wei, X. Wu, Y. Wu, Y. Xiong, Y. Xue, D.M. York, S. Zhao, Q. Zhu, and P.A. Kollman (2023), Amber 2023, University of California, San Francisco.
